# The College of Nursing (Limited by Guarantee)

**Published:** 1921-03-05

**Authors:** 


### THE COLLEGE OF NURSING

**(Limited by Guarantee.)**

### What the Local Centres are Doing.

### LONDON CENTRE.

(Secretary, Miss Bompas, 7 Henrietta Street, W. 1.)

Thursday, March 10, 8 p.m.—The last lecture of the session will be given at the Rooms of the Medical Society, 11 Chandos Street, W. 1. Subject, "The Vote, and what it has done for Women." Speaker, Miss Eleanor Rathbone, C.C., J.P., President National Union of Societies for Equal Citizenship. Members are asked to keep this date free and bring their friends, so that the meeting may be a good one. Admission to members free; non-Centre members, 6d.

Thursday, March 17, 8 p.m.—The London Centre will give a Musical At Home at the Club Rooms, 7 Henrietta Street, W. 1. It is hoped that all new members of the Centre will be able to come so that the Executive may have the pleasure of meeting them.

### Meeting to Discuss Nominations.

Despite earlier and contrary expectations, the numerously attended meeting of the London Centre, which was held on Monday evening, February 28, in the Governors' Hall of St. Thomas's Hospital, agreed, on the recommendation of the Executive Committee, not to take any further steps to nominate official candidates in the coming elections for the Council. The feeling appeared to be that a policy of official nominations—anyhow this year, when it may not have had sufficient consideration—might tend to hamper the freedom of the individual voter, and would place at a heavy and possibly unfair disadvantage a candidate who may not be put forward officially, yet who in every respect may be an ideal Council member. The meeting was, however, warned by Miss Gibson, the Chairman of the Executive Committee, that no candidate has a chance of being elected unless she is well supported by the Centres or by other organisations, and that it behoves every member of the College to work hard in order to secure the election of the candidate whom she wishes to see returned. The present needs of the Council are for more representation of Poor-Law infirmaries, both London and provincial, private nurses, and small hospitals, and members should use all their efforts to secure more adequate representation of these interests. The policy of the Executive Committee, as expressed by Miss Gibson at Monday's meeting, is that it recommends the support of the candidates who came before the Centre that evening, but it feels very strongly that the London Centre members must be free to vote for whom each likes.

The Executive Committee will give the members a further opportunity of discussing the policy of a postal ballot in connection with the eight candidates nominated by Centre members.

The meeting was presided over by Miss Cox-Davies, and after formal business and the announcement of Dame Sidney Browne's confirmed decision to retire from the presidency of the Centre, though continuing, as a member of the Council, to take the keenest interest in the Centre's work, a letter was read from Miss Sordy, Matron of Queen Mary's Hospital, expressing her intention, owing to the number of candidates nominated at the last meeting, to stand aside as an official candidate, though she proposes to allow herself to stand for nomination independently of the Centre, being convinced it is desirable to have on the Council someone who can represent small general and special hospitals. She would, however, like to nominate Mr. C. E. Leo Lyle, M.P., in her place. He worked hard for the profession in connection with Nurses' Registration, and has its interests closely at heart.

The introduction of the candidates nominated by various members at the last Centre meeting, and others, then took place. Lady Cowdray, who had been so nominated, was unable to be present through absence from England; but Miss Cox-Davies explained her reasons for nominating Lady Cowdray, who, she feels, both by her knowledge and interest in nursing, her shrewd judgment and business capacity, is well qualified to become a most useful Council member.

#### The Candidates and their Aims.

The candidates each addressed the meeting, being limited to five minutes. The manner and matter of the address of each was of a remarkably high order. Miss Volta Billing, matron of the Torbay Hospital, Torquay, represents the West Country as well as the interests of nurses working in isolated moorland districts. Miss Edmunds, sister-tutor of St. James's Infirmary, Balham, a member, she said, of the "comparatively young" generation of nurses. If elected she hopes, with her Matron's help, to be able to attend a reasonable number of Council meetings. She stands for fuller teaching, practical and theoretical, for the working nurse, a shorter working day, and adequate salaries, those for probationers being kept as small as possible. Miss Amy Hughes, who is retiring from the Council and offers herself for re-election, stands for the fuller recognition and more just treatment of the district nurse, her work for whom since 1885 is well known. Miss Macdonald, a nurse, now training for welfare work, stands for improved economic conditions for the nursing profession, an eight-hour day on the College plan, and a College superannuation and unemployment scheme. A body, she said, of 20,000 members should surely be able to accomplish that measure of protection. Miss Macdonald further emphasised the importance of the work of a nurse as a citizen. Miss Masters, matron of the Leicester Poor-law Infirmary, stands for Poor-law nursing in the Midlands, and has had twenty-one years' experience. She considers the Poor-law nurse has been much misunderstood in the past. If elected, Miss Masters hopes to be able to make an average number of attendances at Council meetings. Miss Burgess, matron of Crumpsall Infirmary and a member of the East Lancs Centre, spoke in warm support of Miss Masters' nomination. Miss Redl, matron of Brompton Hospital, stands for small and special hospitals, with experience as matron of an affiliated hospital recognised by the College; Brompton Hospital has had a scheme of affiliation since 1890. Miss Redl has done, Miss Cox-Davies stated, yeoman service for the College since its foundation by means of actual and continued service in the work of registration. Miss Spackman, matron of the National Hospital, Queen Square, stands for special hospitals and nurse-masseuses; she desires to see a standardised training for nurses and a scheme for the pensioning of nurses in old age.

Further items on the agenda were a resolution, proposed by Miss Hillyers in a thoughtful paper, and seconded by Miss Bowdler, that the London Centre shall provide for a scholarship to qualify a student as sister-tutor at King's College for Women, and that £150 from the London Centre shall be allocated for this purpose, Miss Cowlin proposed an amendment, which was seconded by Miss Copeman, limiting this undertaking to the year 1921-22, and the resolution so amended was passed. Miss Sheriff MacGregor spoke to the meeting on the necessity for propaganda both by nurses amongst each other and by bringing the work of nurses to the knowledge of girls now being educated. A question concerning the Cowdray Club and the opening of its doors by election to members of other professions than nursing elicited the assurance from Miss Gibson that it was certain that Lady Cowdray would not allow the interests of nurses to suffer; Lady Cowdray had stated, said Miss Cox-Davies, that nurses could always feel that the Cowdray Club was essentially their own.

### SHEFFIELD CENTRE.

(Hon. Sec., Mrs. Barnes, 34 Wilkinson Street.)

Thursday, March 10.—Dr. Egerton Williams on "Inspection in Relation to Common Infectious Diseases" in the General Lecture Room of the University at 6 p.m.

